# Two-dimensional symbiotic solitons and vortices in binary condensates with attractive cross-species interaction

**DOI:** 10.1038/srep34847

**Published:** 2016-10-05

**Authors:** Xuekai Ma, Rodislav Driben, Boris A. Malomed, Torsten Meier, Stefan Schumacher

**Affiliations:** 1 Department of Physics and Center for Optoelectronics and Photonics Paderborn (CeOPP), Universität Paderborn, Warburger Strasse 100, 33098 Paderborn, Germany; 2 Department of Physical Electronics, School of Electrical Engineering, Faculty of Engineering, Tel Aviv University, Tel Aviv 69978, Israel

## Abstract

We consider a two-dimensional (2D) two-component spinor system with cubic attraction between the components and intra-species self-repulsion, which may be realized in atomic Bose-Einstein condensates, as well as in a quasi-equilibrium condensate of microcavity polaritons. Including a 2D spatially periodic potential, which is necessary for the stabilization of the system against the critical collapse, we use detailed numerical calculations and an analytical variational approximation (VA) to predict the existence and stability of several types of 2D *symbiotic solitons* in the spinor system. Stability ranges are found for symmetric and asymmetric symbiotic fundamental solitons and vortices, including *hidden-vorticity* (HV) modes, with opposite vorticities in the two components. The VA produces exceptionally accurate predictions for the fundamental solitons and vortices. The fundamental solitons, both symmetric and asymmetric ones, are *completely stable*, in either case when they exist as gap solitons or regular ones. The symmetric and asymmetric vortices are stable if the inter-component attraction is stronger than the intra-species repulsion, while the HV modes have their stability region in the opposite case.

Multidimensional localized modes in nonlinear dispersive media, which are usually considered as solitons, is a topic of great interest in many areas of physics^1^,^2^,^3^,^4^,^5^,^6^,^7^,^8^,^9^,^10^, especially in nonlinear photonics and in the studies of matter waves in Bose-Einstein condensates (BECs). In addition to their significance to fundamental studies, two- and three-dimensional (2D and 3D) solitons offer potential applications, such as the use of spatiotemporal optical solitons as bit carriers in data-processing schemes^11^ and the design of matter-wave interferometers using 3D matter-wave solitons^12^,^13^,^14^,^15^,^16^.

Unlike 1D solitons, which are usually stable objects^17^,^18^, stability is a major issue for their 2D and 3D counterparts. The ubiquitous cubic self-focusing nonlinearity, which readily produces formal 2D and 3D soliton solutions, gives rise to the wave collapse (*critical collapse* in 2D and *supercritical collapse* in 3D)^19^,^20^,^21^,^22^,^23^, which entirely destabilizes the respective soliton families. For this reason, the first example of solitons which was predicted in nonlinear optics, *viz*., the *Townes solitons*^24^, i.e., 2D self-trapped modes supported by a cubic self-focusing nonlinearity, are subject to the instability caused by the critical collapse, which is why they have not been created in experiment. Multidimensional solitons with intrinsic vorticity (vortex rings), which also formally exist in cubic media (in particular, vortex counterparts of the Townes solitons^25^), are subject to an even stronger splitting instability initiated by azimuthal perturbations^1^.

Thus, the stabilization of multidimensional fundamental and vortex solitons is an issue of great importance in various physical settings. In a 2D geometry with cubic self-focusing nonlinearity, the stabilizing factor must break the specific scale invariance, which underlies the critical collapse. The most straightforward possibility for that is provided by the addition of a spatially periodic (lattice) potential, which fixes a particular spatial scale (the lattice period). As a result, it was predicted that such potentials provide for the stabilization of both fundamental and vortex solitons^26^,^27^,^28^,^29^,^30^,^31^,^32^,^33^,^34^,^35^. In most cases, stable vortices are created in the form of a ring-shaped chain of local density peaks, with the vorticity carried by the superimposed phase profile which features a phase circulation of 2*πS*, with integer *S* being the respective topological charge.

In many cases, the natural interactions in the underlying physical media are repulsive, hence they cannot create regular solitons, which play the role of the ground state in the physical system, although it is possible to create gap solitons as a result of the interplay of the lattice potential and a repulsive nonlinearity^36^,^37^,^38^,^39^,^40^. In particular, inter-atomic forces in bosonic gases are normally repulsive^41^, as well as the exciton-exciton interactions in polariton condensates in semiconductor microcavities^42^,^43^,^44^,^45^.

The overall repulsive interaction may be switched into attraction by means of a Feshbach resonance (FR), in bosonic gases^46^,^47^,^48^,^49^ and polariton condensates^50^ alike. The FR applies to the inter-component interactions in binary (spinor or pseudo-spinor) condensates^51^,^52^,^53^. This suggests a new approach to the creation of solitons in spinor condensates: while each component features self-repulsion, the FR-induced attraction between them makes it possible to create *symbiotic solitons*, supported solely by the attraction between the two components, which may even overcome the intrinsic repulsion in each of them^54^,^55^. In particular, for microcavity polaritons this condition may be satisfied in a narrow spectral range, as explained in more detail below. The formation of localized symbiotic modes in the presence of a lattice potential was studied too, but only in 1D settings^56^,^57^.

The objective of the present work is to develop the concept of symbiotic solitons for 2D spinor condensates with an inter-component cubic attraction and intra-species repulsion. To secure the stability of the 2D solitons against the critical collapse, the system must include a lattice potential, which can be easily implemented in experiments for both atomic BEC (as an optical lattice)^41^ and polariton condensates^58^ (in the latter work, the lattice was used for the experimental creation of 2D exciton-polariton solitons).

The results presented below are obtained by means of a combination of an analytical variational approximation (VA) and a systematic numerical investigation of the existence, stability, and dynamics of the solitons. We find that the VA provides very good accuracy in predicting fundamental and vortex solitons, both symmetric and asymmetric ones, with respect to the two components. We also consider self-trapped modes with *hidden vorticity* (HV), i.e., a soliton with opposite signs of the vorticity in its two components^59^. The solution families include both gap solitons and regular ones, as concerns their placing relative to the bandgap spectrum of the linearized system. It is found that, in the presence of a lattice, all the fundamental solitons are *completely stable* solutions, while the vortices have a stability region when the cross-component attraction is stronger than the intra-species repulsion. The HV modes may be stable in the opposite case.

It is further worth noting that if the filling factor of the lattice is too small or the lattice potential is too weak, 2D solitons supported by the cubic nonlinearity with either attractive or repulsive sign suffer a delocalization transition, i.e., they cannot exist as stationary states trapped by the lattice^60^. In this work, we do not aim to exactly identify delocalization boundaries.

## Results

### The model

The dynamics of both two-component atomic BEC and coherent microcavity polariton gases (with losses appropriately compensated by gain), are modeled by the symmetric pair of coupled nonlinear Schrödinger equations^41^,^42^,


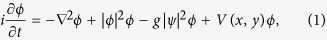



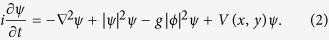


Here *ϕ*(*x*, *y*, *t*) and *ψ*(*x*, *y*, *t*) are the coherent fields of the two components, with ∇^2^ ≡ ∂^2^/∂*x*^2^ + ∂^2^/∂*y*^2^ being the 2D Laplacian and *V*(*x*, *y*) an effective lattice potential. It is assumed, as noted above, that the self-interaction of each component is repulsive, while the cross-interaction is attractive, accounted for by *g* > 0; another mechanism that may give rise to effective attraction in the polariton fluid was proposed^61^. The equations are written in the scaled form, so that the coefficients in front of the Laplacian and self-repulsion terms are normalized to unity. Equal coefficients in front of the Laplacians in both equations imply that the spinor describes a pair of different hyperfine states of the same atom in the bosonic gas. The same symmetry refers to the two spin states of excitons in the polariton gas^62^.

The lattice potential is taken in the usual form, *V* = −[cos(2*πx*/*P*) + cos(2*πy*/*P*)], with spatial period *P*. Below, generic numerical results are adequately presented for *P* = 10. The depth of the potential, *V*_max_ − *V*_min_ = 4, is fixed by means of the remaining scaling invariance. At the origin, *x* = *y* = 0, where the center of the soliton will be placed, the lattice potential has a local minimum. It is easy to check that the shape of the lattice different from quadratic, adopted here (e.g., hexagonal, radial, or anisotropic) will not essentially affect the results^30^.

In our analysis, we numerically sought for stationary localized solutions of Eqs (1) and (2), in the form of


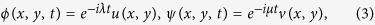


with corresponding chemical potentials *λ* and *μ* and real-valued functions *u* and *v* obeying the stationary equations,


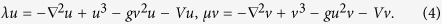


Numerical solutions were constructed on a finite-size grid that was sufficiently large to avoid influence of boundaries on the localized solutions. Stationary solutions were found starting from a localized input, using the imaginary-time propagation method^63^,^64^. The stability of these stationary solutions is then tested by simulations of perturbed evolution of Eqs (1) and (2) in real time.

### Symmetric solitons and vortices

First, we look for symmetric modes with identical stationary wave functions *u* and *v* of the two components, with equal norms


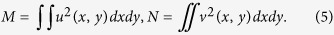


In this symmetric case, the overall nonlinearity present in Eqs (1) and (2) can be characterized by a single effective coefficient *g* − 1. Thus, *g* > 1 makes the effective nonlinearity self-attractive. For *g* < 1 the strength of the repulsive inter-component interaction is larger than the attractive cross-component interaction and thus the net effect in this case is an overall defocusing nonlinearity. Note that the net nonlinearity vanishes in the symmetric case for *g* = 1.

**Figure 1:**
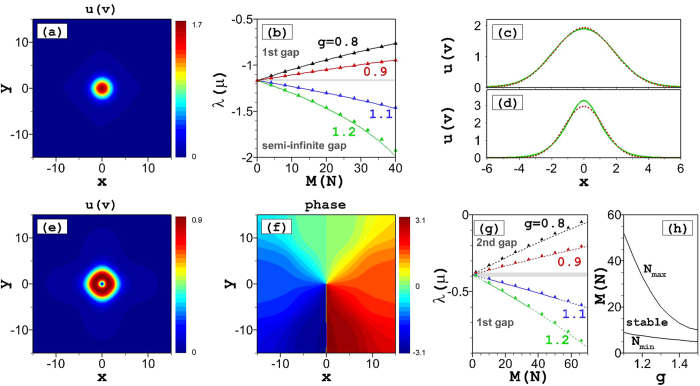
Symmetric solitons and vortices. (**a**) The amplitude distribution in a stable symmetric fundamental soliton, for *g* = 1.1 and *M* = 20. (**b**) Dependence of the norm (*M* ≡ *N*) on the chemical potential (*λ* ≡ *μ*) for the family of fundamental symmetric solitons at different values of the cross-attraction coefficient, *g*. Solid lines depict stable numerically found solutions, while triangles represent the corresponding results produced by the VA as per Eqs (10)–(13). (**c**,**d**) Comparison of numerically found (solid lines) and VA-predicted (dashed lines) profiles of the fundamental symmetric solitons at *g* = 0.8, *M* = 40 (**c**) and *g* = 1.2, *M* = 40 (**d**). Panels (e) and (f) display, respectively the amplitude and phase distributions in a numerically found stable symmetric vortex solitons for *g* = 1.1 and *M* = 20. (**g**) Dependence of the norm (*M* ≡ *N*) on the chemical potential (*λ* ≡ *μ*) for the symmetric vortex solitons for different values of *g*. Solid and dashed lines represent stable and unstable vortices, respectively, while triangles depict the corresponding VA results, as per Eqs (16)–(19). At *g* > 1, the vortices are stable in a finite interval of values of the norm, see Eq. (6). (**h**) Dependence of the stability boundaries of vortices, *N*_max_ and *N*_min_, on *g*. The gray regions in (**b**,**g**) represent the (narrow) first and second bands of the lattice-potential spectrum, respectively.

Numerical results for the symmetric solutions are summarized in Fig. 1. We have found that the symmetric fundamental solitons [see a typical example in Fig. 1(a)] are stable in their *entire existence region*, as shown by Fig. 1(b). This conclusion complies with the fact that, for *g* > 1 and *g* < 1, the *N*(*μ*) dependences obey, severally, the Vakhitov-Kolokolov (VK) and anti-VK criteria, i.e., *dN*/*dμ* < 0 and *dN*/*dμ* > 0, which are necessary, although not sufficient, stability conditions for solitons supported, respectively, by attractive^22^,^65^ and repulsive^66^ nonlinearities.

In fact, the fundamental solitons found at *g* < 1, which correspond to the effective repulsive nonlinearity, are gap solitons^36^. It can be readily checked that values of *λ* ≡ *μ* in Fig. 1(b), corresponding to *g* < 1, fall into the first finite bandgap of the spectrum of the linearized symmetric Eq. (4), while those corresponding to *g* > 1 belong to the semi-infinite gap. These gaps are separated by the (first) narrow band, which is displayed in Fig. 1(b) by a gray-shaded stripe. Unlike the fundamental solitons, the vortices corresponding to *g* > 1 and *g* < 1 fall, respectively, into the first and second finite bandgaps, which are separated by the narrow second band, as shown in Fig. 1(g). It is usually assumed that gap solitons have a loosely bound shape, with many inner oscillations^36^. Nevertheless, they may also feature a tightly bound shape, similar to that of regular solitons, and in that case they can be effectively fitted by means of the VA^39^,^67^, similar to what occurs in the present system.

Also included in Fig. 1(b,c) are the analytical results produced by the VA for the fundamental solitons, as detailed in the Methods Section below. The VA-predicted results agree extremely well with their numerical counterparts (very small deviations are observed for stronger nonlinearity and/or larger norms).

While, as mentioned above, lattice potentials often create vortex states with a complex structure, composed of four density peaks for the sates with vorticity *S* = ±1^26^, simple *crater-shaped* stable vortices may be found too^68^. Symmetric crater-shaped vortices have been found in the present case too, with a typical example displayed in Fig. 1(e,f). As Fig. 1(g) shows, the version of the VA developed for the vortex solitons (see Methods Section for details) also provides a very good accuracy, in comparison with the numerical findings.

As said above, all vortices (here, we consider only those with *S* = ±1) have been found in the first and second finite bandgaps (but not in the semi-infinite gap). The vortices are unstable for *g* < 1, when they belong to the second bandgap. On the other hand, Fig. 1(g) demonstrates that families of the symmetric vortex solitons are stable at *g* > 1 in the first gap, in a finite interval of


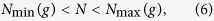


where *N*_max_ decreases with the increase of *g*, see Fig. 1(h). Direct simulations demonstrate that unstable vortices spontaneously evolve into robust randomly vibrating single- or multi-peak patterns, which are asymmetric with respect to the two components.

### Asymmetric solitons and vortices

**Figure 2:**
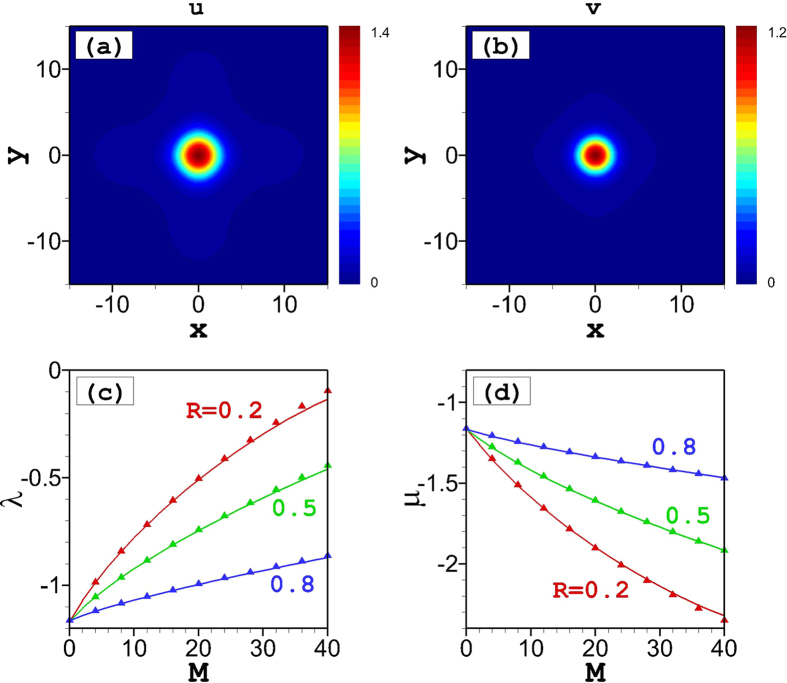
Asymmetric solitons. Amplitude distributions of the *u*- (**a**) and *v*- (**b**) components of the stable asymmetric fundamental soliton with *g* = 1 and *N* = 10, *M* = 20 (i.e., the asymmetry ratio is *R* = 0.5). Panels (c) and (d) display dependences between the larger norm (*M*) and the two chemical potentials, *λ* and *μ*, respectively [see Eqs (5), (12) and (13)], for different values of the asymmetric ratio, *R*, at *g* = 1. Solid lines represent stable numerical solutions, and triangles depict the VA-predicted analytical results from Eqs (10)–(13).

Asymmetric solitons, with unequal *u* and *v* components, form an especially interesting class of modes in the present model. We start their consideration with the case of *g* = 1, in which symmetric solitons cannot exist. Basic results for this case are displayed in Fig. 2. Note that the width of the profile with the larger norm [Fig. 2(a)] is larger than that of its counterpart [Fig. 2(b)] with smaller norm. For a quantitative analysis we define the asymmetry ratio, *R* ≡ *N*/*M*. Then, with *g* = 1 and the asymmetry ratio 0 < *R* < 1, i.e., roughly speaking, |*ψ*|^2^ > |*ϕ*|^2^, Eqs (1) and (2) suggest that the defocusing and focusing nonlinearities dominate in the former and latter equations, respectively. This conclusion is also confirmed by the behavior of the chemical potentials *λ* and *μ*, cf. their behavior in Fig. 2 with that observed in Fig. 1(b) for *g* < 1 and *g* > 1, respectively. Thus, asymmetric solitons with *g* = 1 may be considered as bound states of gap solitons and regular ones, i.e., complexes of the *semi-gap* type^56^, the respective chemical potentials, *λ* and *μ*, falling, respectively, into the first finite gap and semi-infinite one.

The analytical results the VA produces for these asymmetric modes are also found to be in excellent agreement with the numerical findings, as can be seen from the comparison of the analytical and numerical results for varying chemical potentials *λ* and *μ* in Fig. 2(c,d). A detailed comparison (not shown here in detail) demonstrates that, as the *u* component becomes broader with decrease of the asymmetry ratio *R*, this component is more strongly affected by the underlying lattice potential, which slightly worsens the VA accuracy for stronger nonlinearities in the limit of *R* → 0.

Similar to their symmetric counterparts, it has been found that the asymmetric fundamental solitons are stable in their *entire existence region*. Intuitively, this conclusion agrees with the fact that the *λ*(*M*) and *μ*(*M*) dependencies in Fig. 2(c,d) satisfy the anti-VK and VK criteria, respectively.

**Figure 3:**
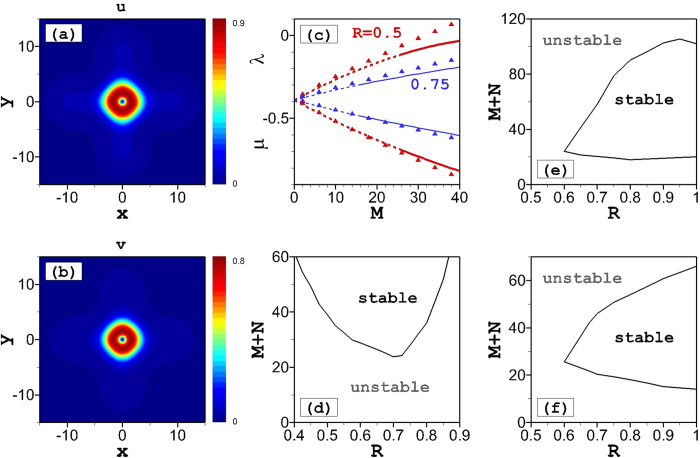
Asymmetric vortices. Amplitude distributions of the *u*- (**a**) and *v*- (**b**) components of the asymmetric (*R* = 0.5) vortex soliton at *g* = 1 and *M* = 20. (**c**) Dependence of the norm (*M*) on the chemical potentials (*μ* and *λ*) of the two components of the vortex for different asymmetry ratios at *g* = 1. Solid and dashed lines denote stable and instable solutions, respectively, while triangles represent the VA predictions, as per Eqs (16)–(19). Regions of stability and instability for the vortices are shown for *g* = 1 in (**d**), *g* = 1.1 in (**e**), and *g* = 1.2 in (**f**).

For asymmetric vortices, the spatial profiles of the *u* and *v* components feature small differences, as seen in Fig. 3(a,b), while the phase distributions are virtually identical in both components, provided that they carry the same vorticity, *S* = +1 or −1, in both components (not shown here). Similar to what is shown above for the fundamental solitons in the case of *g* = 1, when the solutions are asymmetric (*M* ≠ *N*), the repulsive and attractive interactions dominate in the different components, resulting in different dependence between the chemical potentials and the norm, as shown in Fig. 3(c).

The comparison of the VA for asymmetric vortices and numerical results is shown in Fig. 3(c). In the asymmetric case, the vortex component (*u*) with a larger norm is broader than its counterpart with a smaller norm (*v*). Also in this case, due to the influence of the underlying lattice, the agreement of the exact numerical solution with the simplest vortex ansatz, given by Eq. (14), gets worse with increasing vortex size. Therefore, the agreement of the VA and numerical results for the *v* component is better than for the *u* component, see Fig. 3(c). Generally, the size of the vortices is larger than that of the fundamental solitons, therefore the overall agreement of the variational and numerical results for asymmetric vortices in Fig. 3(c) is somewhat poorer than for the asymmetric fundamental solitons in Fig. 2(c,d).

Figure 3(d) shows stability and instability regions for the vortices, depending on the asymmetry ratio, for *g* = 1. At *R* → 1, the vortices become unstable, as the nonlinearity effectively disappears when the solutions approach the symmetric case with *g* = 1. At *R* → 0, the model reduces to a single-component system with a defocusing nonlinearity. The smallest norm for stable vortices is found around *R* = 0.7.

Figure 3(e) shows an example of the stability region for vortices for *g* > 1, *viz*., with *g* = 1.1. Starting from the symmetric case with *R* = 1, the stability range initially slightly widens with the increase of the asymmetry (decrease of *R*). Then, the stability region gradually decreases with further decrease of *R*, until all stable solutions disappear at *R* < 0.6. For *g* = 1.2, as shown in Fig. 3(f), the stability region decreases monotonously with increasing asymmetry (decrease of *R*), again shrinking to nil at *R* ≈ 0.6. At *g* > 1, smaller asymmetry ratio *R* implies domination of the attractive nonlinearity in Eq. (2), eventually leading to an instability of the *v* component. It is worth noting that the fact that vortices are unstable for larger norms in Fig. 3(e,f) is in contrast to the results for *g* = 1, shown in Fig. 3(d). Roughly speaking, the stability regions for *g* > 1, shown in Fig. 3(e,f) are rotated by 90° in comparison with their counterpart shown in Fig. 3(d) for *g* = 1. At *g* < 1, all asymmetric vortices are unstable, similar to what is stated above for the symmetric ones.

### Hidden-vorticity (HV) modes

**Figure 4:**
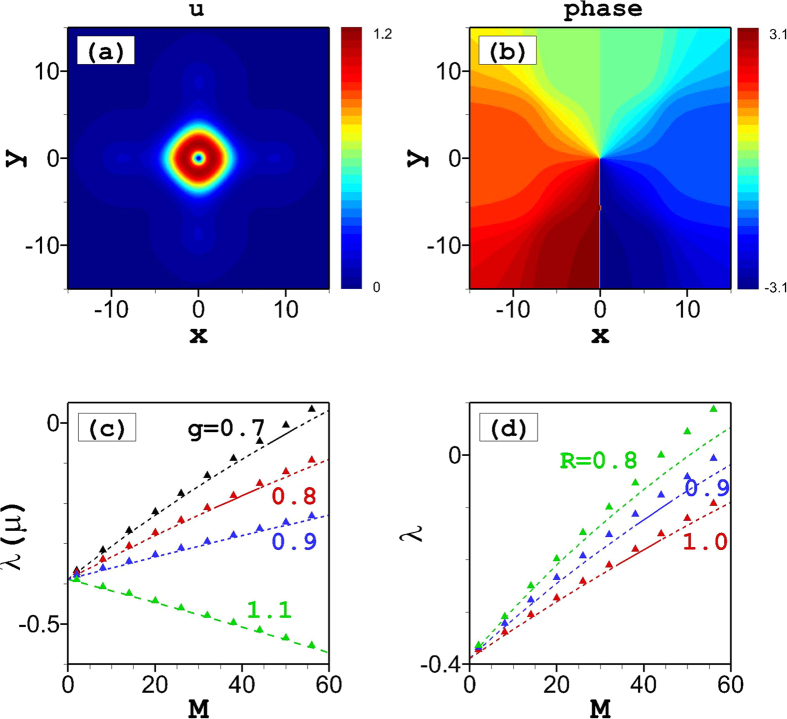
Hidden vorticity modes. (**a**) Amplitude and (**b**) phase distributions of symmetric (*R* = 1) stationary solutions for the *u*-component of an vortex-antivortex pair at *g* = 0.8 and *M* = 35. Dependences of the norm (*M*) on the chemical potential (*λ*) of vortex-antivortex pairs for (**c**) different nonlinearity at *R* = 1 and (**d**) different asymmetric ratio at *g* = 0.8. Solid lines are stable numerical solutions, while dashed lines are unstable solutions. Triangles depict analytical results from Eqs (16)–(19).

For the HV vortex states, in which the two components have opposite vorticities, results are summarized in Fig. 4. The vorticities of the two components are *S* = +1 and −1, corresponding to the phase maps shown in Figs 4(b) and 1(f), respectively. In the symmetric case (*M* = *N*), both components have the same spatial profiles, as shown in Fig. 4(a). In this case, the mode carries zero angular momentum, therefore it is called the HV state^59^.

We have found that the vortex-antivortex pairs are unstable for the overall-focusing nonlinear system, with *g* > 1, but a remarkable fact is that they may be *stable* for the defocusing system, with *g* < 1, as shown in Fig. 4(c). Recall that all states with explicit vorticity, i.e., *S* = 1 in both components, are entirely unstable at *g* < 1, as shown above. We have also found finite stability ranges for the vortex-antivortex pairs at *g* < 1 in the weakly asymmetric case, with asymmetry ratios *R* slightly smaller than 1, as shown in Fig. 4(d). If the norm difference between the two components becomes too large, the vortex-antivortex pairs become unstable.

The VA results for the vortex-antivortex pairs are given by the same equation, Eqs (18) and (19), as for their vortex-vortex counterparts. The analytical results agree very well with the numerical finding when *M* = *N*, see Fig. 4(c). For *M* ≠ *N*, the difference between the analytical and numerical results becomes more apparent due to the larger size of the vortex components, as seen in Fig. 4(d).

## Discussion

We have numerically and theoretically investigated the existence and stability of symbiotic solitons and vortices in the 2D two-component spinor system with repulsive intra-species and attractive cross-component interactions, in the presence of an underlying lattice potential. We have found that the fundamental solitons are *always stable*, in both symmetric and asymmetric cases (equal or different norms of the two components). Vortex solitons with the same sign of the vorticity in both components are stable when the attractive cross-component interaction is stronger than the intra-species repulsion. On the other hand, the modes with hidden vorticity, i.e., opposite signs of the vorticity in the two components, can be stable only when the repulsive interactions dominate. These novel types of self-trapped modes are of interest for the understanding of general principles of pattern formation in 2D nonlinear settings, and they can be realized in physical systems. In atomic gases, the inter-particle interaction can be finely tuned in the vicinity of a FR (Feshbach resonance)^46^, using the strongly dispersive nature of the scattering length near the two-particle resonance (a negative scattering length is associated with an attractive interaction).

This FR control may also be realized for microcavity polaritons, where the frequency dependence of the scattering matrix element has been analyzed in detail for excitations which are placed spectrally below the fundamental exciton resonance^69^,^70^. In a narrow spectral range close to the two-particle resonance associated with the formation of a bound biexciton state, the role of the attractive cross-component interaction can be finely tuned so that it may dominate over the repulsive inter-component interaction. We note that in this spectral range, in addition to the intrinsic loss rate for polaritons, the system will also suffer excitation-induced losses (a similar side effect of the FR is known in atomic BECs). However, these losses may be compensated in the presence of an exciton reservoir, or by an external pump laser, which makes it possible to maintain the quasi-stationary behavior. In this case, the stationary modes predicted in the present work should be observable in semiconductor microcavities.

There remain a number of points for extension of the work, such as hybrid bound states, with a fundamental self-trapped state in one component, and a vortex in the other. A challenging problem is the consideration of a three-dimensional version of the present system, which may be realized in an atomic BEC.

## Methods

### The variational approximation (VA)

The Lagrangian of the stationary equations (4) is


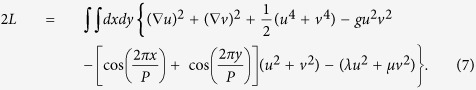


For fundamental solitons, the simplest Gaussian ansatz is adopted, with amplitudes *A*, *B* and radial widths *a*, *b*:


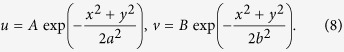


This ansatz produces the following norms, Eq. (5), in the two components: *M* = *πA*^2^*a*^2^, and *N* = *πB*^2^*b*^2^. In the asymmetric case, *M* ≠ *N*, we define the asymmetry ratio *R* = *N*/*M* as noted above, restricting it to 0 < *R* ≤ 1. The substitution of ansatz (8) into Lagrangian (7) and subsequent integration yields the following effective Lagrangian:


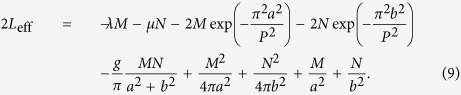


The first pair of the variational equation following from Eq. (9) makes it possible to express *a*^2^ and *b*^2^ in terms of *M* and *N*: ∂*L*_eff_/∂(*a*^2^) = ∂*L*_eff_/∂(*b*^2^) = 0, i.e.,


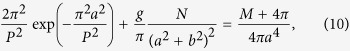



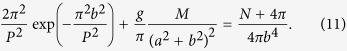


For given *M* and *N*, *a*^2^ and *b*^2^ can be found by means of a numerical solutions of the algebraic system (10) and (11). The chemical potentials, *λ* and *μ*, do not appear in Eqs (10) and (11). They are produced by the second pair of the variational equations: ∂*L*_eff_/∂*M* = ∂*L*_eff_/∂*N* = 0, i.e.,


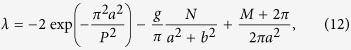



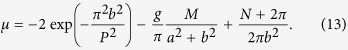


The first objective of the VA is, for given *g*, to identify a region in the plane of (*M*, *N*) where the numerical solution of Eqs (10) and (11) produces physically relevant solutions, with *a*^2^, *b*^2^ > 0.

Similarly to the approach for the fundamental solitons outlined above, for vortices we adopt a natural generalization of the Gaussian ansatz:


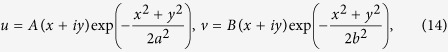


whose norms are *M* = *πA*^2^*a*^4^ and *N* = *πB*^2^*b*^4^. Substituting ansatz (14) into Lagrangian (7), the following effective Lagrangian is obtained, cf. Eq. (9):


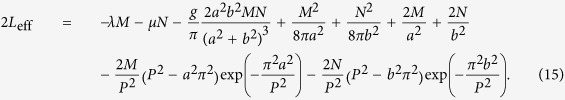


Then, the variational equations for *a*^2^ and *b*^2^ are produced as ∂*L*_eff_/∂(*a*^2^) = ∂*L*_eff_/∂(*b*^2^) = 0, i.e.,


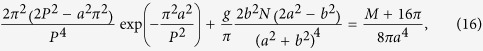



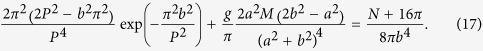


Finally, the chemical potentials for the two components of the vortex are produced according by the remaining variational equations, ∂*L*_eff_/∂*M* = ∂*L*_eff_/∂*N* = 0, i.e.,


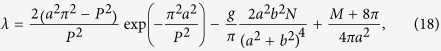



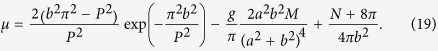


## Additional Information

**How to cite this article**: Ma, X. *et al*. Two-dimensional symbiotic solitons and vortices in binary condensates with attractive cross-species interaction. *Sci. Rep.* **6**, 34847; doi: 10.1038/srep34847 (2016).

## References

[b1] Malomed B. A., Mihalache D., Wise F. & Torner L. Spatiotemporal optical solitons. J. Optics B: Quant. Semicl. Opt. 7, R53–R72 (2005).

[b2] Desyatnikov A. S., Torner L. & Kivshar Y. S. Optical vortices and vortex solitons. Progr. Opt. 47, 1 (2005).

[b3] Mihalache D. Linear and nonlinear light bullets: recent theoretical and experimental studies. Rom. J. Phys. 57, 352–371 (2012).

[b4] Ostrovskaya E. A., Abdullaev J., Desyatnikov A. S., Fraser M. D. & Kivshar Y. S. Dissipative solitons and vortices in polariton Bose-Einstein condensates. Phys. Rev. A 86, 013636 (2012).

[b5] Sich M. . Observation of bright polariton solitons in a semiconductor microcavity. Nature Photon. 6, 50 (2012).

[b6] Amo A. . A. Polariton Superfluids Reveal Quantum Hydrodynamic Solitons. Science 332, 1167 (2011). 21636766 10.1126/science.1202307

[b7] Egorov O. A., Skryabin D. V., Yulin A. V. & Lederer F. Bright Cavity Polariton Solitons. Phys. Rev. Lett. 102, 153904 (2009). 19518634 10.1103/PhysRevLett.102.153904

[b8] Lagoudakis K. G. . Quantized vortices in an exciton-polariton condensate. Nature Phys. 4, 706 (2008).

[b9] Kuopanportti P., Huhtamäki J. A. M. & Möttönen M. Exotic vortex lattices in two-species Bose-Einstein condensates. Phys. Rev. A 85, 043613 (2012).

[b10] Kuopanportti P., Orlova N. V. & Milosevic M. V. Ground-state multiquantum vortices in rotating two-species superfluids. Phys. Rev. A 91, 043605 (2015).

[b11] McLeod R., Wagner K. & Blair S. (3+1)-dimensional optical soliton dragging logic. Phys. Rev. A 52, 3254 (1995). 9912613 10.1103/physreva.52.3254

[b12] Martin A. D. & Ruostekoski J. Quantum dynamics of atomic bright solitons under splitting and recollision, and implications for interferometry. New J. Phys. 14, 043040 (2012).

[b13] Cuevas J., Kevrekidis P. G., Malomed B. A., Dyke P. & Hulet R. G. Interactions of solitons with a Gaussian barrier: Splitting and recombination in quasi-1D and 3D. New J. Phys. 15, 063006 (2013).

[b14] Nguyen J. H. V., Dyke P., Luo D., Malomed B. A. & Hulet R. G. Collisions of matter-wave solitons. Nature Phys. 10, 918–922 (2014).

[b15] McDonald G. D. . Bright solitonic matter-wave interferometer. Phys. Rev. Lett. 113, 013002 (2014). 25032924 10.1103/PhysRevLett.113.013002

[b16] Sakaguchi H. & Malomed B. A. Matter-wave soliton interferometer based on a nonlinear splitter. New J. Phys. 18, 025020 (2016).

[b17] Kivshar Y. S. & Agrawal G. P. Optical Solitons: From Fibers to Photonic Crystals (Academic Press, San Diego, 2003).

[b18] Dauxois T. & Peyrard M. Physics of Solitons (Cambridge University Press: Cambridge, UK, 2006).

[b19] Bergé L. Wave collapse in physics: principles and applications to light and plasma waves. Phys. Rep. 303, 259–370 (1998).

[b20] Kuznetsov E. A. & Dias F. Bifurcations of solitons and their stability. Phys. Rep. 507, 43–105 (2011).

[b21] Fibich G. The Nonlinear Schrödinger Equation: Singular Solutions and Optical Collapse (Springer: Heidelberg, 2015).

[b22] Bergé L. Wave collapse in physics: principles and applications to light and plasma waves. Phys. Rep. 303, 259 (1998).

[b23] Kuznetsov E. A. & Dias F. Bifurcations of solitons and their stability. Phys. Rep. 507, 43 (2011).

[b24] Chiao R. Y., Garmire E. & Townes C. H. Self-trapping of optical beams. Phys. Rev. Lett. 13, 479 (1964).

[b25] Kruglov V. I., Logvin Yu. A. & Volkov V. M. The theory of spiral laser beams in nonlinear media. J. Mod. Opt. 39, 2277–2291 (1992).

[b26] Baizakov B. B., Malomed B. A. & Salerno M. Multidimensional solitons in periodic potentials. Europhys. Lett. 63, 642 (2003).

[b27] Yang J. & Musslimani Z. H. Fundamental and vortex solitons in a two-dimensional optical lattice. Opt. Lett. 28, 2094 (2003). 14587826 10.1364/ol.28.002094

[b28] Baizakov B. B., Malomed B. A. & Salerno M. Multidimensional solitons in a low-dimensional periodic potential. Phys. Rev. A 70, 053613 (2004).

[b29] Mihalache D. . Stable three-dimensional spatiotemporal solitons in a two-dimensional photonic lattice. Phys. Rev. E 70, 055603(R) (2004). 10.1103/PhysRevE.70.055603 15600685

[b30] Baizakov B. B., Salerno M. & Malomed B. A. Multidimensional solitons and vortices in periodic potentials. In: Nonlinear Waves: Classical and Quantum Aspects (ed. by Abdullaev F. Kh. & Konotop V. V.) 61–80 (Kluwer Academic Publishers: Dordrecht, 2004).

[b31] Kartashov Y. V., Vysloukh V. A. & Torner L. Rotary solitons in Bessel optical lattices. Phys. Rev. Lett. 93, 093904 (2004). 15447104 10.1103/PhysRevLett.93.093904

[b32] Kartashov Y. V., Vysloukh V. A. & Torner L. Stable ring-profile vortex solitons in Bessel optical lattices. Phys. Rev. Lett. 94, 043902 (2005). 15783560 10.1103/PhysRevLett.94.043902

[b33] Baizakov B. B., Malomed B. A. & Salerno M. Matter-wave solitons in radially periodic potentials. Phys. Rev. E 74, 066615 (2006). 10.1103/PhysRevE.74.066615 17280170

[b34] Driben R., Malomed B. A., Gubeskys A. & Zyss J. Cubic-quintic solitons in the checkerboard potential. Phys. Rev. E 76, 066604 (2007). 10.1103/PhysRevE.76.066604 18233934

[b35] Mayteevarunyoo T., Malomed B. A., Baizakov B. B. & Salerno M. Matter-wave vortices and solitons in anisotropic optical lattices. Physica D 238, 1439–1448 (2009).

[b36] Baizakov B. B. & Konotop V. V. and Salerno, M. Regular spatial structures in arrays of Bose-Einstein condensates induced by modulational instability. J. Phys. B: At. Mol. Opt. Phys. 35, 5105 (2002).

[b37] Konotop V. V. & Salerno M. Modulational instability in Bose-Einstein condensates in optical lattices. Phys. Rev. A 65, 021602 (2002).

[b38] Louis P. J. Y., Ostrovskaya E. A., Savage C. M. & Kivshar Y. S. Bose-Einstein condensates in optical lattices: Band-gap structure and solitons. Phys. Rev. A 67, 013602 (2003).

[b39] Sakaguchi H. & Malomed B. A. Two-dimensional loosely and tightly bound solitons in optical lattices and inverted traps. J. Phys. B 37, 2225 (2004).

[b40] Ostrovskaya E. A. & Kivshar Y. S. Matter-Wave Gap Vortices in Optical Lattices. Phys. Rev. Lett. 93, 160405 (2004). 15524961 10.1103/PhysRevLett.93.160405

[b41] Stoof H. T. C., Gubbels K. B. & Dickerscheid D. B. M. Ultracold Quantum Fields (Springer: Dordrecht, 2009).

[b42] Kavokin A., Baumberg J. J., Malpuech G. & Laussy F. P. Microcavities (Oxford University Press: Oxford, 2007).

[b43] Keeling J., Marchetti F. M., Szymanska M. H. & Littlewood P. B. Collective coherence in planar semiconductor microcavities. Semicond. Sci. Technol. 22, R1 (2007).

[b44] Shelykh I. A., Kavokin A. V., Rubo Y. G., Liew T. C. H. & Malpuech G. Polariton polarization-sensitive phenomena in planar semiconductor microcavities. Semicond. Sci. Technol. 25, 013001 (2010).

[b45] Carusotto I. & Ciuti C. Quantum fluids of light. Rev. Mod. Phys. 85, 299 (2013).

[b46] Pollack S. E. . Extreme tunability of interactions in a ^7^Li Bose-Einstein condensate. Phys. Rev. Lett. 102, 090402 (2009). 19392500 10.1103/PhysRevLett.102.090402

[b47] Chin C., Grimm R., Julienne P. & Tiesinga E. Feshbach resonances in ultracold gases. Rev. Mod. Phys. 82, 1225 (2010).

[b48] Yamazaki R., Taie S., Sugawa S. & Takahashi Y. Submicron Spatial Modulation of an Interatomic Interaction in a Bose-Einstein Condensate. Phys. Rev. Lett. 105, 050405 (2010). 20867900 10.1103/PhysRevLett.105.050405

[b49] Yan M., DeSalvo B. J., Ramachandhran B., Pu H. & Killian T. C. Controlling Condensate Collapse and Expansion with an Optical Feshbach Resonance. Phys. Rev. Lett. 110, 123201 (2013). 25166803 10.1103/PhysRevLett.110.123201

[b50] Takemura N., Trebaol S., Wouters M., Portella-Oberli M. T. & Deveaud B. Polaritonic Feshbach resonance. Nature Phys. 10, 500 (2014).

[b51] Zhang P., Naidon P. & Ueda M. Independent control of scattering lengths in multicomponent quantum gases. Phys. Rev. Lett. 103, 133202 (2009). 19905512 10.1103/PhysRevLett.103.133202

[b52] de Forges de Parny L., Rousseau V. G. & Roscilde T. Feshbach-stabilized insulator of bosons in optical lattices. Phys. Rev. Lett. 114, 195302 (2015). 26024178 10.1103/PhysRevLett.114.195302

[b53] Wang F., Li X., Xiong D. & Wang D. A double species ^23^Na and ^87^Rb Bose-Einstein condensate with tunable miscibility via an interspecies Feshbach resonance. J. Phys. B: At. Mol. Opt. Phys. 49, 015302 (2016).

[b54] Pérez-Garca V. M. & Beitia J. B. Symbiotic solitons in heteronuclear multicomponent Bose-Einstein condensates. Phys. Rev. A 72, 033620 (2005).

[b55] Adhikari S. K. Bright solitons in coupled defocusing NLS equation supported by coupling: Application to Bose-Einstein condensation. Phys. Lett. A 346, 179 (2005).

[b56] Adhikari S. K. & Malomed B. A. Symbiotic gap and semigap solitons in Bose-Einstein condensates. Phys. Rev. A 77, 023607 (2008).

[b57] Roeksabutr A., Mayteevarunyoo T. & Malomed B. A. Symbiotic two-component gap solitons. Opt. Exp. 20, 24559–24574 (2012). 10.1364/OE.20.024559 23187219

[b58] Cerda-Méndez E. A. . Exciton-polariton gap solitons in two-dimensional lattices. Phys. Rev. Lett. 111, 146401 (2013). 24138259 10.1103/PhysRevLett.111.146401

[b59] Brtka M., Gammal A. & Malomed B. A. Hidden vorticity in binary Bose-Einstein condensates. Phys. Rev. A 82, 053610 (2010).

[b60] Baizakov B. & Salerno M. Delocalizing transition of multidimensional solitons in Bose-Einstein condensates. Phys. Rev. A 69, 013602 (2004).

[b61] Dominici L. . Real-space collapse of a polariton condensate. Nature Commun. 6, 8993 (2015). 26634817 10.1038/ncomms9993 PMC4686858

[b62] Lagoudakis K. G. . Observation of Half-Quantum Vortices in an Exciton-Polariton Condensate. Science 326, 974 (2009). 19965506 10.1126/science.1177980

[b63] Chiofalo M. L., Succi S. & Tosi M. P. Ground state of trapped interacting Bose-Einstein condensates by an explicit imaginary-time algorithm. Phys. Rev. E 62, 7438 (2000). 10.1103/physreve.62.7438 11102106

[b64] Antoine X., Bao M. & Besse C. Computational methods for the dynamics of the nonlinear Schrödinger/Gross–Pitaevskii equations. Comp. Phys. Commun. 184, 2621 (2013).

[b65] Vakhitov N. G. & Kolokolov A. A. Stationary solutions of the wave equation in a medium with nonlinearity saturation. Radiophys. Quant. Electron. 16, 783 (1973).

[b66] Sakaguchi H. & Malomed B. A. Solitons in combined linear and nonlinear lattice potentials. Phys. Rev. A 81, 013624 (2010).

[b67] Adhikari S. & Malomed B. A. Gap solitons in a model of a superfluid fermion gas in optical lattices. Physica D 238, 1402–1412 (2009).

[b68] Sakaguchi H. & Malomed B.A. Higher-order vortex solitons, multipoles, and supervortices on a square optical lattice. Europhys. Lett. 72, 698–704 (2005).

[b69] Kwong N. H., Takayama R., Rumyantsev I., Kuwata-Gonokami M. & Binder R. Third-order exciton-correlation and nonlinear cavity-polariton effects in semiconductor microcavities. Phys. Rev. Lett. 64, 045316 (2001).

[b70] Schumacher S., Kwong N. H. & Binder R. Influence of exciton-exciton correlations on the polarization characteristics of polariton amplification in semiconductor microcavities. Phys. Rev. B 76, 245324 (2007).

